# Supplementary figures and images for: Designing a computer-assisted diagnosis system for cardiomegaly detection and radiology report generation
Source: PLOS Digit Health. 2025 May 20;4(5):e0000835. doi: 10.1371/journal.pdig.0000835 (PMC12091825; doi:10.1371/journal.pdig.0000835)

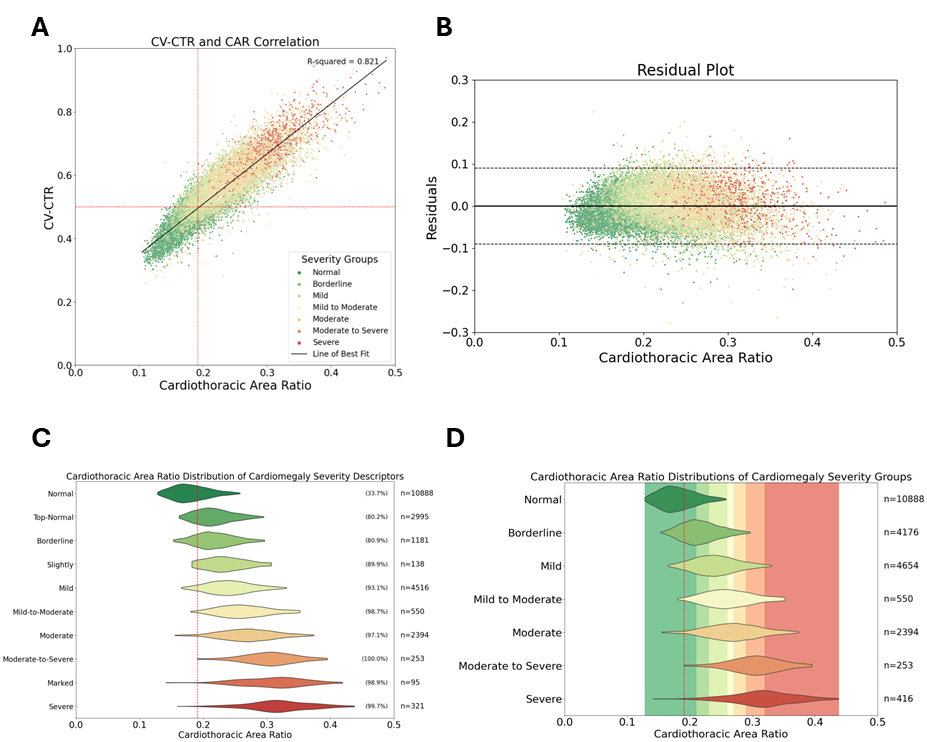

Supplement: S1 Fig — Data-driven severity classification based on natural language extracted labels and computer vision-based cardiothoracic area ratio (CAR) calculation. (A) CV-CAR vs CV-CTR with the 7 severity groups from reports indicated by colours. The horizontal red dashed line represents CV-CTR = 0.50 (common threshold for cardiomegaly), the vertical red dashed line represents the CAR value for the lowest 1% of all cardiomegaly cases (from mild to severe), with 99% of cardiomegaly cases to be found above this threshold. (B) Residual plot based on the line of best fit. The parallel black dashed lines represents the interval where 90% of residuals reside. (C) Violin plot of the CV-CAR for all categories prior to merging categories. (D) Violin plot of the CV-CAR after consolidation of categories. (C and D) The red dashed line represents the 1% CAR value threshold for cardiomegaly cases [from mild to severe, as in (A)]. (TIF) [file pdig.0000835.s001.tif]
