# Supplementary material for: Designing a computer-assisted diagnosis system for cardiomegaly detection and radiology report generation
Source: PLOS Digit Health. 2025 May 20;4(5):e0000835. doi: 10.1371/journal.pdig.0000835 (PMC12091825; doi:10.1371/journal.pdig.0000835)
Supplement: Attachment — Submitted filename: PDIG-D-24-00379_revision_responsetoreviewers.pdf [file pdig.0000835.s003.pdf]

Reviewer #1: Overall, the manuscript presents a promising approach to utilizing computer-assisted diagnosis (CAD) for cardiomegaly detection and radiology report generation, achieving impressive accuracy. The research is technically sound and addresses an important problem in radiology, particularly in reducing radiologist workloads.

1. Technical Soundness and Data Support:
The study demonstrates a well-structured methodology with clear data to support its findings. The use of the MIMIC-CXR dataset is appropriate, and the system's performance, particularly in terms of accuracy (95.2%) and report generation consistency, is commendable. However, some additional details on how the dataset inconsistencies were corrected would enhance transparency.

RESPONSE: We thank the reviewer for their comments. We have addressed these in the manuscript as well, but to summarise the two key decisions to correct inconsistencies: 1) If an image is classified as abnormal in terms of quality, it will not be used at any stage of the cardiomegaly detection process, as shown in Fig. 2.2). For the corrected view, if the predicted view is PA/AP, the image is retained for training or testing; otherwise (prediction of a lateral view), the image is excluded from this study. The results obtained from the prediction model are reported in the metadata file, following the same structure as MIMIC, with additional columns for our system's predictions.

2. Statistical Analysis:
While the manuscript focuses primarily on the model's accuracy, there is limited mention of detailed statistical analysis. It would be helpful to include more information on the statistical methods used to validate the model's performance, such as confidence intervals or statistical significance testing, to ensure robustness.

RESPONSE: We thank the reviewer for pointing this out. We report the results of three methods. The first two methods involve preprocessing using a neural network: classifying images as normal vs. abnormal and determining the predicted view. In Fig. 3 we reported the median [interquartile range (IQR)] across 10 models initialised with different random states, demonstrating that our performance is not merely due to the chance of favourable weight initialisation. The confusion matrix also includes the worst and best performance for reference. The third method is for cardiomegaly prediction, we originally included AUC and accuracy metrics; we have now added precision and recall (as also requested by reviewer 3). This method does not rely on a neural network or deep learning model; therefore, repeating the analysis on the same test set will yield identical results. We believe that the current test set is sufficiently large to represent the method's performance across diverse examples, however we acknowledge that given that we only use the MIMIC-CXR data it remains to be tested on different datasets before we can make a claim around its generalisability.

3. Data Availability:
The use of the publicly available MIMIC-CXR dataset complies with PLOS's data availability policy. However, the authors should ensure that all data underlying their findings, including any processed or additional datasets, are available in a public repository or provided as supplementary material.

RESPONSE: Our metadata file, which includes all images checked for quality and view, is provided in the supplementary materials. The code used to create the models is available on GitHub (https://github.com/jz4520/CXR-Based-Cardiomegaly-CAD-). We have also added

information the make-up of our test set, to allow direct comparisons of other work to ours in future.

4. Language and Presentation:
The manuscript is generally well-written and presented in standard English, with only minor grammatical or typographical errors that can be addressed during revisions. The content is clear and understandable, making it accessible to a broad audience.

RESPONSE: We thank the reviewer for their thorough review and understanding of our aim to make the manuscript broadly accessible. We have gone over the manuscript during this revision and corrected all grammatical and typographical errors we have come across.

5. Improvements and Future Directions:
The authors mention future improvements to the text generation aspect of the CAD system. It would be beneficial to include more specific details on how these enhancements will be approached. Additionally, a more in-depth explanation of how the system corrects inconsistencies in the dataset would strengthen the manuscript.

RESPONSE: We thank the reviewer for this comment, which is also echoed by comments from reviewer 3. We have made clear that our system is not meant to be used in clinic at this time. In the discussion we outline limitations of the current system that would need to be addressed in future. Additionally, we added further details on the quality assessment part of this work.

6. Recommendation:
Overall, I recommend minor revisions to address the points mentioned above, particularly the need for more detail on the statistical analysis and future improvements.

RESPONSE: We thank the reviewer for their thorough review and we hope our revisions outlined above and below, in response to other reviewers, have suitably addressed the suggestions made.

Reviewer #2: The study presents a compelling, well-structured CAD system that addresses a significant need in radiology by automating cardiomegaly detection and report generation. The use of a large, publicly available dataset (MIMIC-CXR) and the system's high accuracy rates make it a strong contribution to the field. The authors have effectively combined computer vision with NLP to create a practical solution for radiologists, supported by validation from certified radiologists.

Additional clarity on how explainability was ensured in decision-making, particularly in areas where the model diverged from human interpretations, would be beneficial.

RESPONSE: We thank the reviewer for their comments and feedback on the original manuscript. We have added to the methods and discussion on the limitations of the current model, including where human readers disagreed with the CAD system in terms of the severity of cardiomegaly and report text generation (through inter-annotation agreement rate). For the quality assessment and view correction models, we manually reviewed the predictions, and no major discrepancies were observed between the models' predictions and our assessments.

Reviewer #3: The present paper proposes building a system in which x-ray images are processed and put through a pipeline to classify cardiomegaly and then produce a written radiology report. The novelty and effectiveness of the proposed system are unclear from the paper. The lack of clarity is in part due to the presentation and writing of the paper. The paper may be suitable for publication after the following major issues are addressed.

RESPONSE: We thank the reviewer for their constructive comments and have outlined our responses and revisions made below. We expanded on the contribution/novelty here and made this clearer in the manuscript. We did not want to raise any false beliefs that our system was in clinical use, or that we advocate for it to be used in its current state.

Major comments:

The novelty of the paper is lacking, and the objective is not clear. The abstract and introduction need to be rewritten to lay out the structure and define the novelty. The methodology and results need to be rewritten to present the information linearly and logically.

RESPONSE: We have rewritten the objectives of our work to make clearer the goal and novelty of the work. We also rewrote sections of the manuscript, addressing comments below, to improve the flow.

The paper fails to compare with prior work.

RESPONSE: We had included a comparison with several other published papers, as commented on by the reviewer below. We included only manuscripts that focussed on the same topic, and for which classification performance was available. To the best of our knowledge, no other study has been published to use computer vision model to predict the severity of cardiomegaly, subsequently use this information to generate human-like text and compare this with human radiologists' assessments. We have mentioned in the limitations the difficulty of comparing between different studies, and we disclose in the Supporting Materials the exact test set we used to allow others to compare directly against our work in future.

The clinical application is unclear. For example, does the method work for a screening application with an asymptomatic population? Why would people be screened for cardiomegaly and not also for pneumonia and other diseases? If only for cardiomegaly, then how are incidental findings handled?

RESPONSE: We have clarified the context of our work; we used a single dataset and did not (at this time) test it in other datasets. This work was not applied in clinic (we have clarified this in relation to a comment below). Patients would certainly be assessed for other conditions, therefore we evaluated the co-occurrence of cardiomegaly with other co-morbidities and conditions/observations. We showed that within the reports, cardiomegaly typically only appears by itself within a sentence of the report (no other term co-occurs with it consistently). For example, pneumonia also often appears by itself (or together with mentions of opacities). Other cases, such as focal consolidation, pleural effusion and pneumothorax are often mentioned in the same sentence when all findings are negative. For this reason we chose cardiomegaly as the exemplar due to the fact it is reported individually, so is less affected by other findings. Moreover, other related work has predominantly treated cardiomegaly as a binary classification task, whereas we predict the severity.

When Resnet is trained, it is not clear what "filtered images" are.

RESPONSE: We thank the reviewer for this comment, while it was evident to us what we meant by this we noticed we used the same terminology for different aspects. Initially ‘filtered’ could refer to either ‘poor quality’ images (being ‘filtered out’) or those that were of sufficient quality to be analysed by the view correction model (filtering the good ones). We have revised this throughout the document and no longer use the word filtered/filtering for this.

The segmentation algorithm should be compared to outlines of the heart created by radiologists.

RESPONSE: The segmentation algorithm has previously been validated, here we used its outputs. We have compared the derived predictions of cardiomegaly with radiologists’ assessments, demonstrating higher levels of agreement between the human readers and our prediction than between human readers.

The performance of the model is evaluated solely on the MIMIC-CXR dataset. There is no external validation using independent datasets to assess the generalizability of the model. Without testing on other datasets, it is unclear whether the model's performance holds in different clinical settings or with data from other sources.

RESPONSE: The reviewer is correct, and we have amended the discussion to highlight these as one limitation of the current work. Within those limitations, we did explore the comorbidities that often co-occur with cardiomegaly, and evaluated whether there exists a difference between the CV-CTR across different conditions/findings (Fig. 6 A and B).

The manuscript claims a state-of-the-art accuracy of 95.2% for binary cardiomegaly classification. However, this claim is based on comparisons with studies that may have used different datasets, evaluation metrics, or methodologies. Without benchmarking the model against existing methods using standardized datasets and protocols, the claim is not substantiated.

RESPONSE: It is indeed true that direct comparisons of performance is challenging to make due to different datasets being used. We have scaled back our claim and provided context of the comparisons, as well as emphasising the difference in test datasets. Due to other authors not disclosing their training, validation and test set splits, or in many cases the data itself, we have added this information in the Supplementary Materials to allow others in future to compare their results against ours in a direct comparison.

Why is Natural Language Processing used to generate the report instead of a rule-based system?

RESPONSE: Reports are written by human radiologists in different styles. The same finding can be described in different ways. We use NLP to emulate this to show there are differences. Our radiologists were not aware that some of the reports contained sentences that were machine generated, whereas they would have surely spotted a rule-based system when comparing this to human written reports. We have elaborated on this in the discussion.

The paper spends extensive time discussing the errors in the dataset and how they were identified (Table 2). However, the denominator of the total inaccuracies is not identified, indicating that there are even more errors in the dataset.

RESPONSE: We chose to represent the errors in percentages rather than full numbers for brevity, we have amended this to make clear that we reviewed all reports in the dataset for which errors were identified and also we labelled all images for which the view was not available.

ROC, precision, and recall should be reported. Estimates of error should be calculated and reported with all performance metrics.

RESPONSE: We have now included these metrics where appropriate, such as for ResNet models, in response to this comment (echoed by reviewer 1).

The paper's statistical methods have several concerns. The use of multiple t-tests with Šidák correction to identify statistically synonymous severity descriptors is problematic. Data skewness and unequal variances violate key t-test assumptions. Assigning threshold values by selecting the highest p-value from t-tests at incremental intervals is not a standard method for cut-off determination in continuous data. This approach increases the risk of Type I errors and does not adequately handle issues from multiple comparisons.

RESPONSE: We agree that parametric t-test may not have been the most appropriate, and upon revising we have first used the Shapiro-Wilk test for normality (most groups are indeed not normally distributed) and subsequently used Mann-Whitney U tests – see the table below for comparison between methods. However, despite using a different statistical test, the outcome remains the same in that cases with descriptors of "slightly" and "mild" are not significantly different, and neither are "moderate-to-severe" and "marked", and "marked" and "severe". The original grouping of severity descriptors was therefore not altered – we have revised the methods to include details on the different tests used.

Severity adjective	Shapiro-Wilk test p-value
normal	1.68e-26
borderline	1.84e-4
slightly	1.39e-1
top-normal	1.55e-12
mild	6.61e-9
mild-to-moderate	8.13e-2
moderate	2.37e-10
moderate-to-severe	1.78e-3
severe	1.24e-4
marked	1.11e-2

Comparison	T-test p-value	Mann-Whitney U-test p-value	Result
normal vs top-normal	<1e-16	<1e-16	
Top-normal vs borderline	8.74e-1	7.59e-1	n.s.
borderline vs slightly	3.67e-7	1.99e-8	
slightly vs mild	4.23e-1	3.60e-1	n.s.
mild vs mild-to-moderate	<1e-16	<1e-16	
mild-to-moderate vs moderate	1.21e-2	3.50e-4	
moderate vs moderate-to-severe	<1e-16	<1e-16	
moderate-to-severe vs marked	1.23e-1	2.19e-1	n.s.
marked vs severe	1.00	9.68e-1	n.s.
moderate-to-severe vs severe	2.95e-4	5.21e-7	

The methodology should be rewritten to enable reproducibility. Currently, methodology and implementation details are missing or unclear. Unnecessary implementation details should be moved to appendix or removed.

RESPONSE: We have added details on methods used and made a GitHub repository with the code used to train and test models (https://github.com/jz4520/CXR-Based-Cardiomegaly-CAD-) to allow reproduction of the results. Moreover, as mentioned above, we have included the details on the data used in our testing phase.

Minor comments:

The use of adjectives and adverbs introduces ambiguity into the paper. The use of compounded sentence structure hinders the readability and clarity of the paper. Adjectives and adverbs should be deleted or replaced with quantifiers. Compound and complex sentence structures with mixed clauses should be simplified and presented linearly. The issue is most evident in the abstract and introduction.

RESPONSE: We have revised sentence structure and word choices in the paper to reduce ambiguity and enhance readability.

Heading and subheadings are overused and misused throughout the paper. The current use of subheadings hinders readability and obscures the continuity and logic between the paragraphs. The number of subheadings should be reduced and the paragraphs should be connected cohesively to each other.

RESPONSE: We have removed all \subsubsection headers in the LaTeX file to improve this.

Figures are misplaced in the middle of paragraphs and not discussed in the paper.

RESPONSE: We used the PLOS LaTeX template and this automatically places the figures close to where they are first mentioned. We have checked and all figures were referenced in the main text.

The input of the dataset into MedQBot may violate the IRB of the dataset.

RESPONSE: We have expanded and rewritten this part to make clear what we did to ensure we adhered to the data sharing agreement. We also emphasise here that we did not supply MedQBot with the dataset as the reviewer may have inadvertently assumed. Instead, we only passed single sentences that were altered by us, only sentences from the findings section, and only sentences that appear across many reports, therefore none of the sentences that were input into MedQBot can be traced back to any specific report. With few-shot learning new sentences were generated in the same style.

The paper briefly mentions future improvements but fails to discuss current limitations in depth, such as the model's inability to handle images with other pathologies that affect heart size or lung fields.

RESPONSE: We have expanded on some of these limitations in the revised discussion.

"Our computer vision model, developed as part of the CAD system, exhibited good 437 performance for binary cardiomegaly classification." "Good" is an unsubstantiated adjective.

RESPONSE: This was substantiated in the third sentence of the same paragraph in the original manuscript where we mention the accuracy in percentage terms. We have revised the passage accordingly.

The discussion should be expanded to include a discussion on the gap between validation metrics and real-world performance.

RESPONSE: We have added this to the limitations section of the discussion.

The paper lacks a discussion on the legal implications of deploying an autonomous CAD system and replacing/augmenting a radiologist in clinical settings, including regulatory approvals and compliance with healthcare laws.

RESPONSE: We would like to make clear that we did not use the system in clinic. As the reviewer will be able to appreciate this process would indeed take several years to conduct. As mentioned above, we have re-angled the paper to make our objectives clearer. We have added to the discussion/conclusion a description of how others can follow our process to locally train algorithms on their data, this will allow fine-tuning models to local data.
