# Supplementary material for: Designing a computer-assisted diagnosis system for cardiomegaly detection and radiology report generation
Source: PLOS Digit Health. 2025 May 20;4(5):e0000835. doi: 10.1371/journal.pdig.0000835 (PMC12091825; doi:10.1371/journal.pdig.0000835)
Supplement: Attachment — Submitted filename: PDIG-D-24-00379_revision2_responsetoreviewers.pdf [file pdig.0000835.s004.pdf]

**Dr Joram M. Posma, *PhD, MSc, B AS, PGDip, MRSC, FHEA***
Senior Lecturer in Biomedical Informatics
Section of Bioinformatics, Division of Systems Medicine, Department of Metabolism, Digestion and Reproduction, Faculty of Medicine, Imperial College London
Hammersmith Campus, Burlington Danes Building, Du Cane Road,
London, W12 0NN, United Kingdom
j.posma11@imperial.ac.uk | https://profiles.imperial.ac.uk/j.posma11

06 March 2025

Ref: PDIG-D-24-00379R1 ("Designing a computer-assisted diagnosis system for cardiomegaly detection and radiology report generation")

**Response to Reviewers**

We thank both reviewers for their reviews and confirming we suitably addressed their technical comments and suggestions.

We outline below the changes made after going through the manuscript line-by-line. Our manuscript was written in LaTeX using Overleaf and as such as cannot provide a 'Tracked Changes' version as with Microsoft Word. For this reason, we submitted the revised document previously (R1) as PDF with all changes highlighted as per the journal guidelines labelled "Revised Manuscript with Track Changes". We regret that it appears the reviewers have not been able to see this document for the revision. We have done the same for this revision, and additionally copy below all sentences that were changed/removed from R1 to R2 indicating the old and new line numbers.

**Original** (abstract): This fatigue can reduce diagnostic accuracy and slow down report generation.
**Revised** (abstract): This fatigue may reduce diagnostic accuracy and slow down report generation.

**Original** (2-4): For over 100 years, chest X-ray (CXR) has remained the primary thoracic imaging diagnostic tool due to its low acquisition time and low cost compared to other imaging modalities, and its high versatility and convenience [1, 2].
**Revised** (2-3): For over a century, chest X-rays (CXR) have been the primary diagnostic tool in thoracic imaging due to their fast acquisition, low cost, and versatility [1, 2].

**Original** (4-6): Thoracic anatomical structures, including the lungs and heart, are visualised and distinguished on a 2D film based on radiodensity variations.
**Revised** (3-5): Thoracic anatomical structures, such as the lungs and heart, are visualised and distinguished on a 2D image through radiodensity variations.

**Original** (15-16): Its short acquisition time has lead of widespread and increased use as primary diagnostic test.
**Revised** (15): CXR's short acquisition time has led to widespread use as a primary diagnostic test.

**Original** (18-19): Combined with severe radiologist shortages, this causes a struggled of meeting the demand of providing reports in a timely manner [10].
**Revised** (17-18): Combined with radiologist shortages, this causes a struggle to meet the demand for timely reports [10].

**Original** (19-21): Radiologists are subject to cognitive biases, distractions, and fatigue negatively affecting their diagnostic acumen [11, 12].
**Revised** (19-20): Radiologists are affected by cognitive biases, distractions, and fatigue, which negatively impact their diagnostic acumen [11, 12].

**Original** (21-23): Despite the technical advancements and error-mitigating strategies developed over the years, the radiology interpretive error rates has remained at 3% to 4% for the past 75 years [11–13].

IMPERIAL

**Dr Joram M. Posma, *PhD, MSc, B AS, PGDip, MRSC, FHEA***
Senior Lecturer in Biomedical Informatics
Section of Bioinformatics, Division of Systems Medicine, Department of Metabolism, Digestion and Reproduction, Faculty of Medicine, Imperial College London
Hammersmith Campus, Burlington Danes Building, Du Cane Road,
London, W12 0NN, United Kingdom
j.posma11@imperial.ac.uk | https://profiles.imperial.ac.uk/j.posma11

**Revised** (20-22): Despite technical advancements and error-mitigating strategies over the past 75 years, radiology interpretive error rates have remained at 3% to 4% [11-13].

**Original** (24-25): To remedy this, recent exploratory efforts have been made to incorporate artificial intelligence (AI) technology into CXR interpretation [5].
**Revised** (23-24): To address this, recent exploratory efforts aim to integrate artificial intelligence (AI) technology into CXR interpretation [5].

**Original** (25-27): The underlying rationale is that, unlike their human counterparts, 'AI readers' are immune to fatigue and distractions.
**Revised** (24-25): The underlying rationale is that 'AI readers', unlike humans, are not affected by fatigue and distractions.

**Original** (27-28): AI may also suffer less from certain types of cognitive biases based on the training methods, allowing for higher accuracy and consistency [5].
**Revised** (25-27): AI can avoid certain cognitive biases due to its training methods, leading to higher accuracy and consistency [5].

**Original** (28-30): Additionally, AI could significantly relieve the burden of radiologists, reducing the backlog and improving patient outcomes [14, 15].
**Revised** (27-28): Furthermore, AI could reduce the workload of radiologists, thereby decreasing backlogs and improving patient outcomes [14, 15].

**Original** (35-36): Whereas, AI-CenterNet CXR [19] was developed as a fully automated system for detecting and localising eight thoracic conditions without human involvement.
**Revised** (33-34): Whereas AI-CenterNet CXR [19] was developed as an automated system for detecting and localising eight thoracic conditions without human involvement.

**Original** (40-41): Our study focuses on developing a proof-of-concept CAD system that integrates the classification aspect with report generation.
**Revised** (38-39): Our study focuses on developing a proof-of-concept CAD system that integrates classification with report generation.

**Original** (42-45): Severe cardiomegaly on CXR is immediately apparent to experienced radiologists on a Posterior-Anterior (PA) CXR, while more ambiguous cases may require the calculation of cardiothoracic ratio (CTR) using a PA CXR (Fig 1).
**Revised** (40-43): Experienced radiologists can quickly identify severe cardiomegaly from a Posterior-Anterior (PA) CXR, while more ambiguous cases may require calculating the cardiothoracic ratio (CTR) using the same PA CXR (Fig 1).

**Original** (46-49): The process can be time-consuming and contain a degree of subjectiveness due to variations in thresholds used and measurements taken, thus may benefit from the implementation of CAD systems.
**Revised** (44-46): The process can be time-consuming and contain a degree of subjectiveness due to variations in thresholds used and measurements made, thus could be improved by using CAD systems.

**Original** (51-53): A recent transfer learning model [24] achieved an AUC of 0.87 for classifying normal and cardiomegaly CXR images, in line with similar approaches [25-27].

IMPERIAL

**Dr Joram M. Posma, *PhD, MSc, B AS, PGDip, MRSC, FHEA***
Senior Lecturer in Biomedical Informatics
Section of Bioinformatics, Division of Systems Medicine, Department of Metabolism, Digestion and Reproduction, Faculty of Medicine, Imperial College London
Hammersmith Campus, Burlington Danes Building, Du Cane Road,
London, W12 0NN, United Kingdom
j.posma11@imperial.ac.uk | https://profiles.imperial.ac.uk/j.posma11

**Revised** (48-51): A recent transfer learning model [24] achieved an Area-Under-the-ROC Curve (AUC) of 0.87 for classifying normal and cardiomegaly CXR images, in line with similar approaches [25–27].

**Original** (55-57): While these models boast higher explainability [29, 30], they may not be as predictive due to the limitation of lack of pixel-level labelled CXR data, restricting model generalisability [24, 30].
**Revised** (52-55): While these models claim higher explainability [29, 30], they may not be as predictive due to the limitation of lack of pixel-level labelled CXR data, restricting model generalisability [24, 30].

**Original** (57-60): In terms of generating realistic reports, the recent advancements in natural language processing (NLP) and large language models (LLM) can be leveraged to produce report sentences based on machine predictions that are styled as those written by human radiologists.
**Revised** (55-57): Recent advancements in natural language processing (NLP) and large language models (LLMs) can be leveraged to generate realistic report sentences that are styled as those written by human radiologists.

**Original** (61-64): However, without the capability of determining the severity of heart enlargement and without evaluation of the quality of such computer-generated reports by human radiologists, it is not possible to assess the true capacity to which these CAD systems could automate cardiomegaly detection and reporting.
**Revised** (57-61): Currently, without the ability to determine the severity of heart enlargement from machine predictions and without evaluating the quality of computer-generated reports by human radiologists, we cannot fully assess how effective these CAD systems are at automating cardiomegaly detection and reporting.

**Original** (69-71): 3) combine the output of the computer vision classification model with large language models to generate plausible medical text
**Revised** (66-68): 3) combine the output of the CV classification model with large language models to generate plausible medical text

**Original** (105-107): All images from p10 and p11 folders were scrutinised manually to identify 'poor quality' images which are those that cannot be properly interpreted by radiologists and would warrant retaking the image.
**Revised** (99-101): All 75,216 images from p10 and p11 folders were scrutinised manually to identify 'poor quality' images which are those that cannot be properly interpreted by radiologists and 100 would warrant retaking the image.

**Original** (107-111): These images contain severe cropping, distortion, and/or absence of thoracic structures. Upon positive identification, these images were used to generate 500 additional 'artificial' poor quality images by applying synthetic and augmentation techniques to imitate common types of poor quality images from normal images (see examples in Fig 3A).
**Revised** (101-105): The 22 identified 'poor quality' images contain severe cropping, distortion, and/or absence of thoracic structures and were used to generate 500 additional 'artificial' poor quality images by applying synthetic and augmentation techniques to imitate common types of poor quality images from normal images (see examples in Fig 3A).

**Original** (128-130): For both models, the training image datasets were randomly split into training and validation sets at a ratio of 80:20, enabling real-time model training performance monitoring.

IMPERIAL

**Dr Joram M. Posma, *PhD, MSc, B AS, PGDip, MRSC, FHEA***
Senior Lecturer in Biomedical Informatics
Section of Bioinformatics, Division of Systems Medicine, Department of Metabolism, Digestion and Reproduction, Faculty of Medicine, Imperial College London
Hammersmith Campus, Burlington Danes Building, Du Cane Road,
London, W12 0NN, United Kingdom
j.posma11@imperial.ac.uk | https://profiles.imperial.ac.uk/j.posma11

**Revised** (127-128): For both models, the training image datasets were randomly split into training and validation sets at a ratio of 80:20 for model training performance monitoring.

**Original** (130-131): The ResNet50 model [37] was loaded without pre-trained weights, and all images were resized to 256x256 pixels.
**Revised** (128-130): The ResNet50 model [37] was loaded without pre-trained weights, and all images were resized to 256 × 256 pixels.

**Original** (131-133): Data augmentation was incorporated into training data loading which randomly introduced contrast jitter and rotations to all images for boosting data variability, model versatility and robustness.
**Revised** (130-132): Data augmentation was incorporated into training data loading which randomly introduced contrast jitter and rotations to all images to increase data variability, model versatility and robustness.

**Original** (174-175): The code for classification models can be found at https://github.com/jz4520/CXR-Based-Cardiomegaly-CAD-.
**Revised** (173-174): The code for classification models can be found at https://github.com/omicsNLP/CXR-Based-Cardiomegaly-CAD.

**Original** (203-204): Comorbidity analysis was performed for each report using annotations of 14 conditions in the CheXpert CSV.
**Revised** (202-203): Comorbidity analysis was performed for each report using NLP annotations of 14 conditions present in the CheXpert CSV.

**Original** (236-238): To evaluate our cardiomegaly CAD pipeline performance, four radiologists (three with 1 to 5 years of experience, and one with 10+ years of experience) were invited to participate in a small-scale assessment using the online Qualtrics questionnaire.
**Revised** (236-238): To evaluate our cardiomegaly CAD pipeline performance, four radiologists (three with 1 to 5 years of experience, and one with 10+ years of experience) were invited to participate in a small-scale assessment using an online Qualtrics questionnaire.

**Original** (243-245): Each item was seen by two radiologists who were blinded to each other's answers and were not informed whether the sentences were machine-generated or from the original MIMIC report.
**Revised** (243-245): Each case was reviewed by two radiologists, who were blinded to each other's responses and unaware of whether the sentences were machine-generated or taken from the original MIMIC report.

**Original** (249-251): The machine-generated cardiomegaly sentences were randomly selected from the pre-curated list as described before based on the predicted severity group by the computer vision model.
**Revised** (249-251): The machine-generated cardiomegaly sentences were randomly selected from the pre-curated list as described before based on the predicted severity group by the CV model.

**Original** (274-276): The MIMIC-CXR dataset is one of the largest publicly available chest X-ray radiology datasets, thus containing a considerable degree of data variability.
**Revised** (273-275): The MIMIC-CXR dataset is one of the largest publicly available chest X-ray radiology datasets and includes considerable data variability.

IMPERIAL

**Dr Joram M. Posma, *PhD, MSc, B AS, PGDip, MRSC, FHEA***
Senior Lecturer in Biomedical Informatics
Section of Bioinformatics, Division of Systems Medicine, Department of Metabolism, Digestion and Reproduction, Faculty of Medicine, Imperial College London
Hammersmith Campus, Burlington Danes Building, Du Cane Road,
London, W12 0NN, United Kingdom
j.posma11@imperial.ac.uk | https://profiles.imperial.ac.uk/j.posma11

**Original** (279-280): Selecting appropriate subsets of a dataset for downstream applications is complicated by a host of factors.
**Revised** (277-278): Selecting appropriate subsets of a dataset for downstream applications is complicated by many factors.

**Original** (280-282): As mentioned before, frontal CXR is best suited for cardiomegaly detection, while lateral images are rarely used and should thus be excluded.
**Revised** (278-280): As mentioned, frontal CXR images are best suited for cardiomegaly detection, while lateral images are rarely used and therefore were excluded.

**Original** (283-284): View labels of images were occasionally missing or incorrect, for instance, by mislabelling a lateral image as PA/AP.
**Revised** (281-282): View labels of images were occasionally missing or incorrect, such as mislabelling a lateral image as frontal.

**Original** (285-286): In addition, a smaller proportion of images suffer from quality issues severe enough to entirely prevent analysis.
**Revised** (282-284): In addition, a smaller proportion of images have quality issues that completely prevent analysis.

**Original** (286-288): To address this, the CAD system needs to first identify and flag unsuitable images to prevent further processing and analysis while alerting human radiologists.
**Revised** (284-285): To address this issue, the CAD system must first identify and flag unsuitable images to avoid further processing and analysis.

**Original** (293-295): Additionally, the model can also be utilised to help correct mislabelling and flag flawed images within the MIMIC-CXR dataset, or potentially any similar CXR datasets.
**Revised** (290-292): Additionally, the model can also help correct mislabelling and identify flawed images in the MIMIC-CXR dataset (see Fig 3A for synthetic training images).

**Original** (296-300): 22 poor quality images were identified from p10 and p11 subfolders and categorised into five groups based on similar features (Fig 3A). For each category, 100 new images were generated with synthetic and augmentation techniques. Together, they constituted an poor quality training subset of 500 images to train the first model to classify normal vs poor quality images.
**Revised** (removed)

**Original** (300-303): This model demonstrated an overall accuracy of 99.2% (AUROC=0.989, precision=0.996, recall=0.995) on a test set with 20 real bad-quality images from the MIMIC-CXR and 1,000 normal images with 85% recall for poor quality class (Fig 3B).
**Revised** (293-295): This model demonstrated an overall accuracy of 99.2% (AUC=0.989, precision=0.996, recall=0.995) on a test set with 20 real poor quality images from the MIMIC-CXR and 1,000 normal images with 85% recall for poor quality class (Fig 3B).

**Original** (303-305): The second model, which differentiates between frontal and lateral CXR, exhibited better performance, with 99.4% accuracy (AUROC=0.996, precision=0.993, recall=0.996) on the test set with 1,000 frontal and lateral images each (Fig 3D).

IMPERIAL

**Dr Joram M. Posma, *PhD, MSc, B AS, PGDip, MRSC, FHEA***
Senior Lecturer in Biomedical Informatics
Section of Bioinformatics, Division of Systems Medicine, Department of Metabolism, Digestion and Reproduction, Faculty of Medicine, Imperial College London
Hammersmith Campus, Burlington Danes Building, Du Cane Road,
London, W12 0NN, United Kingdom
j.posma11@imperial.ac.uk | https://profiles.imperial.ac.uk/j.posma11

**Revised** (296-298): The second model, which differentiates between frontal and lateral CXR, exhibited better performance, with 99.4% accuracy (AUC=0.996, precision=0.993, recall=0.996) on the test set with 1,000 frontal and lateral images each (Fig 3D).

**Original** (314-317): Gaining a comprehensive understanding of the cardiomegaly landscape in the MIMIC-CXR dataset is paramount to CAD development. 'Normal' is the most common descriptor for heart size with 10,883 cases, followed by 'Mild' (4,651 cases), 'Borderline' (4,172 cases) and 'Moderate' (2,392 cases) when describing positive cardiomegaly.
**Revised** (307-309): In MIMIC-CXR, 'Normal' is the most common descriptor for heart size with 10,883 cases, followed by 'Mild' (4,651 cases), 'Borderline' (4,172 cases) and 'Moderate' (2,392 cases) when describing positive cardiomegaly.

**Original** (322-324): For each pair of neighbouring descriptors, Mann-Whitney U-tests were performed to identify statistically synonymous severity descriptors which were then merged to form severity categories.
**Revised** (313-315): For each pair of neighbouring descriptors, Mann-Whitney U-tests were performed to identify statistically synonymous severity descriptors which were then merged to form seven severity categories.

**Original** (331-335): Significant overlaps are observed between the distribution of neighbouring descriptors. To identify statistically synonymous severity descriptors, multiple t-tests were carried out between CTR distributions of each pair of neighbouring descriptors, and any neighbouring descriptors with non-significant differences were merged into groups, forming a total of seven severity categories (Fig 4A).
**Revised** (removed)

**Original** (342-344): Using previously established severity group CV-CTR intervals, a confusion matrix was constructed to evaluate heart size predictions of the model against MIMIC ground truths (Fig 4C).
**Revised** (327-329): Using previously established severity group CV-CTR intervals, a confusion matrix was constructed to evaluate heart size predictions of the model against MIMIC 'ground truth' labels (Fig 4C).

**Original** (344-345): At both ends of the severity spectrum, the model performs relatively well in accurately determining cardiomegaly severity.
**Revised** (329-330): The model effectively assessing cardiomegaly severity at both ends of the severity spectrum.

**Original** (349-351): The severity classification based on CV-CTR is similar to the cardiothoracic area ratio (CAR), defined here as the ratio of the total heart area and lung area as presented in PA CXR images.
**Revised** (334-335): The severity classification based on CV-CTR is similar to the CAR, defined here as the ratio of the total heart area and lung area as presented in PA CXR images.

**Original** (351-354): We found the distribution pattern of CV-CAR to be highly similar to that of CV-CTR, and the two are found to be positively linearly correlated (R2=0.82), with 90% of residuals within ±0.09, and with the CV-CAR showing similar patterns across the 7 severities (Supplementary Materials, Figure 7).
**Revised** (335-336): The distribution pattern of CV-CAR closely resembles that of CV-CTR.

IMPERIAL

**Dr Joram M. Posma, *PhD, MSc, B AS, PGDip, MRSC, FHEA***
Senior Lecturer in Biomedical Informatics
Section of Bioinformatics, Division of Systems Medicine, Department of Metabolism, Digestion and Reproduction, Faculty of Medicine, Imperial College London
Hammersmith Campus, Burlington Danes Building, Du Cane Road,
London, W12 0NN, United Kingdom
j.posma11@imperial.ac.uk | https://profiles.imperial.ac.uk/j.posma11

**Original** (351-354): We found the distribution pattern of CV-CAR to be highly similar to that of CV-CTR, and the two are found to be positively linearly correlated (R2=0.82), with 90% of residuals within ±0.09, and with the CV-CAR showing similar patterns across the 7 severities (Supplementary Materials, Figure 7).
**Revised** (336-337): They are found to be positively correlated (R2=0.82), with 90% of residuals within ±0.09.

**Original** (351-354): We found the distribution pattern of CV-CAR to be highly similar to that of CV-CTR, and the two are found to be positively linearly correlated (R2=0.82), with 90% of residuals within ±0.09, and with the CV-CAR showing similar patterns across the 7 severities (Supplementary Materials, Figure 7).
**Revised** (337-339): Additionally, the CV-CAR exhibits consistent patterns across the 7 severity categories (Supplementary Materials, Figure 7).

**Original** (355-356): Once a severity category prediction has been made by the computer vision model, it is used as an input argument for report sentence generation using LLMs.
**Revised** (340-341): Once a severity category prediction has been made by the CV model, it is used as an input argument for report sentence generation using LLMs.

**Original** (375-376): We evaluated the relationship between other mentions in the reports and cardiomegaly.
**Revised** (360-361): We evaluated the relationship between other conditions in the reports and cardiomegaly.

**Original** (382-385): Regardless of the underlying causes, if left unchecked and unbeknownst to researchers, they could pose significant challenges to ensuring data quality and reliability in future studies utilising the dataset.
**Revised** (367-370): Regardless of the underlying causes, if not addressed and unnoticed by researchers, the quality issues could pose significant challenges to ensure reliability of future studies utilising the dataset.

**Original** (385-386): Current literature involving MIMIC-CXR, however, rarely mentions the dataset quality shortcomings, an odd predicament given the popularity of the dataset.
**Revised** (370-371): Current literature involving the dataset rarely mentions the dataset quality shortcomings, nor do they address it.

**Original** (390-392): Out of five studies published over the past two years involving the use of MIMIC-CXR images for creating deep learning models, none has acknowledged any aspects of quality issues within the dataset [40–44].
**Revised** (374-377): Out of five studies published over the last few years involving the use of MIMIC-CXR images for creating deep learning models [40-44], none has acknowledged any aspects of quality issues within the dataset.

**Original** (393-384): We sought to ascertain the extent of selected quality issues within MIMIC-CXR and rectify them for the benefit of future studies using the same dataset.
**Revised** (378-379): We aimed to identify and address certain quality issues in MIMIC-CXR to assist future studies using the dataset.

IMPERIAL

**Dr Joram M. Posma, *PhD, MSc, B AS, PGDip, MRSC, FHEA***
Senior Lecturer in Biomedical Informatics
Section of Bioinformatics, Division of Systems Medicine, Department of Metabolism, Digestion and Reproduction, Faculty of Medicine, Imperial College London
Hammersmith Campus, Burlington Danes Building, Du Cane Road,
London, W12 0NN, United Kingdom
j.posma11@imperial.ac.uk | https://profiles.imperial.ac.uk/j.posma11

**Original** (394-396): Manual identification of bad-quality images or wrong view labels is possible, but time-consuming considering the overwhelming number of images involved.
**Revised** (379-381): Manual identification of bad-quality images or wrong view labels is possible for smaller datasets, but the volume of images in MIMIC-CXR makes this process practically impossible.

**Original** (416-419): This is unlike other potential use cases such as pneumonia (occuring with mentions of opacities), or focal consolidation, pleural effusion and pneumothorax that are often mentioned in the same sentence when all findings are negative.
**Revised** (401-404): This is unlike other potential use cases such as pneumonia (occurring with mentions of opacities), or focal consolidation, pleural effusion and pneumothorax that are often mentioned in the same sentence when findings for these 3 are negative.

**Original** (419-420): Our computer vision-based model was used to predict the severity of cardiomegaly, and thereby also binary classification.
**Revised** (404-405): Our CV-based model was used to predict the severity of cardiomegaly, and thereby also binary classification.

**Original** (439-441): Furthermore, while these past studies have developed similar CAD systems for binary cardiomegaly detection, few have gone to lengths to implement functions for differentiating between different levels of severity.
**Revised** (424-425): While previous studies have developed similar CAD systems for binary cardiomegaly detection, they have not developed functions that distinguish varying severity levels.

**Original** (Table 3): AUC = Area Under (ROC) Curve.
**Revised** (Table 3): AUC = Area Under the (ROC) Curve.

**Original** (443-446): The current study is unique in that it identified the most common descriptors used, grouped synonyms based on statistical analysis and derived CV-CTR intervals for each severity category, which makes the CAD output more informative and is indispensable for downstream report sentence generation.
**Revised** (427-429): Our work stands out by identifying the most used descriptors, grouping synonyms through statistical analysis, and establishing CV-CTR intervals for each severity category.

**Original** (443-446): The current study is unique in that it identified the most common descriptors used, grouped synonyms based on statistical analysis and derived CV-CTR intervals for each severity category, which makes the CAD output more informative and is indispensable for downstream report sentence generation.
**Revised** (429-430): These advancements make the CAD output more informative and crucial for generating report sentences.

**Original** (451-454): While fluctuations in segmentation and computer vision model performance certainly do contribute to the overlaps, natural subjectivity and personal biases of radiologists are also likely factors.
**Revised** (435-437): While fluctuations in segmentation and CV model performance contribute to the overlaps, the subjective nature and personal biases of radiologists are also likely factors.

**Original** (466-468): In addition, studies have shown that CTR may have limited diagnostic power when compared to cardiac MRI, the gold standard for cardiac chamber enlargement detection [49, 50].

**Dr Joram M. Posma**, ***PhD, MSc, B AS, PGDip, MRSC, FHEA***
Senior Lecturer in Biomedical Informatics
Section of Bioinformatics, Division of Systems Medicine, Department of Metabolism, Digestion and Reproduction, Faculty of Medicine, Imperial College London
Hammersmith Campus, Burlington Danes Building, Du Cane Road,
London, W12 0NN, United Kingdom
j.posma11@imperial.ac.uk | https://profiles.imperial.ac.uk/j.posma11

**Revised** (450-451): CTR has limited diagnostic power when compared to cardiac MRI, the gold standard for cardiac chamber enlargement detection, according to some studies [49, 50].

**Original** (468-469): Therefore, we have explored the CAR as an alternative metric for cardiomegaly detection.
**Revised** (452): Therefore, we investigated the CAR as an alternative metric for cardiomegaly detection.

**Original** (469-471): While not used clinically, the concept considers the definition and presentation of cardiomegaly and has the advantage of considering two dimensions of the heart as opposed to a single dimension used for CTR calculation.
**Revised** (453-455): Although not used clinically, this concept considers the definition and presentation of cardiomegaly by advantageously considering two heart dimensions instead of the single dimension used in CTR calculations.

**Original** (473-476): However, the reliability of the CAD system may be improved by cross-checking CV-CTR and CV-CAR derived from the same images, flagging instances where a significant mismatch is found between predicted severity by both methods.
**Revised** (457-459): However, the CAD system's reliability could be enhanced by cross-checking CV-CTR and CV-CAR from the same images, identifying mismatches in predicted severity between both methods.

**Original** (480-482): This reflected the outcome of the co-occurrence analysis showing a frequent co-occurrence between the two conditions (Fig 6B).
**Revised** (463-465): This reflected the outcome of the Ochiai co-occurrence analysis showing a frequent co-occurrence between the two conditions (Fig 6B).

**Original** (483-484): The inclusion of an automatic report sentence generation module is another feature less commonly found in other cardiomegaly CAD systems.
**Revised** (466-467): The inclusion of an automatic report sentence generation module is another unique feature in a cardiomegaly CAD system.

**Original** (486-487): With radiologists only looking at images for 5-10 seconds [9], this means they spend the majority of their time writing the report.
**Revised** (468-470): Radiologists spend 5-10 seconds on each image [9], highlighting they primarily spend their time writing reports.

**Original** (487-489): Therefore we explored whether we can generate report-style sentences based on the computer vision model output, and had four radiologists assess the text quality.
**Revised** (470-471): Therefore, we explored whether we can generate report-style sentences based on the CV model output, and had four radiologists assess the text quality.

**Original** (489-491): Compared to more diverse and complex conditions, the reporting of cardiomegaly has the advantage of being quite consistent and uncomplicated, which enables LLMs to imitate their style effectively.
**Revised** (472-473): Compared to more diverse and complex conditions, the reporting of cardiomegaly is generally consistent and uncomplicated, enabling LLMs to imitate their style effectively.

IMPERIAL

**Dr Joram M. Posma, *PhD, MSc, B AS, PGDip, MRSC, FHEA***
Senior Lecturer in Biomedical Informatics
Section of Bioinformatics, Division of Systems Medicine, Department of Metabolism, Digestion and Reproduction, Faculty of Medicine, Imperial College London
Hammersmith Campus, Burlington Danes Building, Du Cane Road,
London, W12 0NN, United Kingdom
j.posma11@imperial.ac.uk | https://profiles.imperial.ac.uk/j.posma11

**Original** (493-494): By using few-shot learning or fine-tuning approaches, LLMs can write sentences that mimic the style of a specific radiologist.
**Revised** (475-477): By using few-shot learning or fine-tuning approaches, LLMs can generate sentences that imitate individual radiologists' styles.

**Original** (495-497): Such sentences can then be suggested to the radiologist when writing the report opposed to a rule-based system which may contain sentences that a specific radiologist does not typically use.
**Revised** (477-479): Such sentences can then be suggested to the radiologist when writing the report opposed to a rule-based system which may contain sentences that this radiologist does not typically use.

**Original** (497-499): While this is not the scope of this work, this could be integrated into the local software used to view images and speed up writing reports by suggesting sentences that a radiologist can then add to the report, or edit them.
**Revised** (479-482): While this is not the scope of this work, such integration could be implemented within local software to accelerate report writing by suggesting sentences that a radiologist can either include in their report or edit as needed.

**Original** (499-502): The choice of a radiologist for a specific sentence, whether or not they edit them after, can then also be used as part of a reinforcement learning by human feedback (RLHF) approach to improve such a system.
**Revised** (482-484): The radiologist's decision to use a specific sentence and whether they choose to edit it afterward can also serve as part of a reinforcement learning by human feedback approach to enhance such a system.

**Original** (514-516): In fact, disagreements appear to be mostly linguistic rather than diagnostic, as evidenced by the distribution of reasons for disagreement cited, which can be improved by curating a better set of generated sentences.
**Revised** (496-497): Disagreements are primarily linguistic rather than diagnostic, as evidenced by the reasons for disagreements.

**Original** (514-516): In fact, disagreements appear to be mostly linguistic rather than diagnostic, as evidenced by the distribution of reasons for disagreement cited, which can be improved by curating a better set of generated sentences.
**Revised** (497-498): These can be addressed by improving the quality of generated sentences.

**Original** (518-520): While the preliminary results offer much encouragement, the study remains a proof-of-principle at this stage, owing to the lack of large-scale trials involving clinical validation.
**Revised** (500-501): While the preliminary results are encouraging, the study remains a proof-of-principle at this stage due to the absence of large-scale clinical validations.

**Original** (521-523): While we compare our model to prior work, they did not release their data making it impossible to compare performance directly (or our models' generalisability).
**Revised** (503-505): While we compare our model to prior work, they did not release their data or models, thereby preventing direct comparison or limiting the assessment of our models' generalisability.

IMPERIAL

**Dr Joram M. Posma, *PhD, MSc, B AS, PGDip, MRSC, FHEA***
Senior Lecturer in Biomedical Informatics
Section of Bioinformatics, Division of Systems Medicine, Department of Metabolism, Digestion and Reproduction, Faculty of Medicine, Imperial College London
Hammersmith Campus, Burlington Danes Building, Du Cane Road,
London, W12 0NN, United Kingdom
j.posma11@imperial.ac.uk | https://profiles.imperial.ac.uk/j.posma11

**Original** (523-526): To the best of our knowledge, no other large datasets with English-language reports are publicly available, therefore there may be gap between our reported accuracy and 'real-world' performance (including different co-morbidities and clinical settings) of such an algorithm.
**Revised** (505-506): To the best of our knowledge, no other large datasets with English-language reports are publicly available.

**Original** (523-526): To the best of our knowledge, no other large datasets with English-language reports are publicly available, therefore there may be gap between our reported accuracy and 'real-world' performance (including different co-morbidities and clinical settings) of such an algorithm.
**Revised** (506-508): Therefore, there may be gap between the accuracy we report and 'real-world' performance (including different co-morbidities and clinical settings) of such an algorithm.

**Original** (529-531): Furthermore, the case-by-case performance of the computer vision model is dependent on accurate anatomical segmentations, and as such is vulnerable to errors arising from aberrant segmentations.
**Revised** (511-512): Furthermore, the per-case performance of the CV model relies on accurate anatomical segmentations, making it prone to errors due to aberrant segmentations.

**Original** (531-535): It is especially a concern among CXR images where the cardiac silhouette and/or part of the lungs is obscured by conditions such as pulmonary edema and pleural effusion, which can significantly diminish the reliability of segmentations obtained with algorithms trained with limited data on comorbidities.
**Revised** (512-514): This is particularly concerning in CXR images where either the cardiac silhouette or part of the lungs are obscured by conditions like pulmonary oedema and pleural effusion.

**Original** (531-535): It is especially a concern among CXR images where the cardiac silhouette and/or part of the lungs is obscured by conditions such as pulmonary edema and pleural effusion, which can significantly diminish the reliability of segmentations obtained with algorithms trained with limited data on comorbidities.
**Revised** (514-516): These conditions can reduce the reliability of segmentations from algorithms trained with limited data on comorbidities.

**Original** (536-539): In essence, while the CAD system holds significant potential for clinical deployment if further developed, it is not this study's intention to use it as such in its current state, and thus regulatory frameworks and legal aspects will not be discussed in detail.
**Revised** (517-519): In essence, while the CAD system has considerable potential for clinical deployment with further development, it is not the intention of this study to use it as such in its current state.

**Original** (536-539): In essence, while the CAD system holds significant potential for clinical deployment if further developed, it is not this study's intention to use it as such in its current state, and thus regulatory frameworks and legal aspects will not be discussed in detail.
**Revised** (520): Therefore, regulatory frameworks and legal aspects will not be discussed.

**Original** (564-566): By calculating CTR using a computer vision model from deep learning-derived heart and lung segmentations, high overall accuracy in detecting cardiomegaly and assigning appropriate severity labels is achieved.

IMPERIAL

**Dr Joram M. Posma**, ***PhD, MSc, B AS, PGDip, MRSC, FHEA***
Senior Lecturer in Biomedical Informatics
Section of Bioinformatics, Division of Systems Medicine, Department of Metabolism, Digestion and Reproduction, Faculty of Medicine, Imperial College London
Hammersmith Campus, Burlington Danes Building, Du Cane Road,
London, W12 0NN, United Kingdom
j.posma11@imperial.ac.uk | https://profiles.imperial.ac.uk/j.posma11

**Revised** (545-547): By calculating CTR using a CV model from deep learning-derived heart and lung segmentations, high overall accuracy in detecting cardiomegaly and assigning appropriate severity labels is achieved.

**Original** (566-568): Furthermore, the model is capable of generating human-like, report-styled sentences based on the output of the computer vision model.
**Revised** (547-548): Furthermore, the model is capable of generating human-like, report-styled sentences based on the output of the CV model.

**Original** (568-571): Performance metrics indicate that the ability of the model to generate accurate cardiomegaly report sentences based on PA CXR is likely to be non-inferior to human radiologists, although larger trials are necessary to confirm this judgment.
**Revised** (548-550): Performance metrics suggest that the model's ability to produce accurate cardiomegaly report sentences using PA CXR is likely non-inferior to human radiologists.

**Original** (568-571): Performance metrics indicate that the ability of the model to generate accurate cardiomegaly report sentences based on PA CXR is likely to be non-inferior to human radiologists, although larger trials are necessary to confirm this judgment.
**Revised** (550-551): Thus, further studies are needed for confirmation.

**Original** (571-573): While the current model remains a proof-of-concept and is not deployed in clinic, it can serve as a framework for others to further optimise/fine-tune the models and test its performance with local data.
**Revised** (551-553): While the current model remains a proof-of-concept and is not deployed in clinic, it can serve as a framework for others to further optimise or fine-tune these models and evaluate their performance on local data.

**Original** (573-575): When validated in such a manner, the novel CAD system could represent an accessible and cost-effective framework to offer radiologists a second perspective to validate their own assessments.
**Revised** (553-555): Upon validation in this way, the novel CAD system could represent an accessible and cost-effective framework providing radiologists with a second opinion for validating their assessments.
